# Images - Extra (too many) carpal bones in Larsen's syndrome

**DOI:** 10.4103/0971-3026.50837

**Published:** 2009-05

**Authors:** Mukund D Rahalkar, Anand M Rahalkar, Sandeep A Patwardhan

**Affiliations:** Department of Radiology, Sahyadri Speciality Hospital, Plot No. 30-C, Erandawane, Pune - 411 004, Maharashtra, India; 1 Department of Orthopaedics, Sahyadri Speciality Hospital, Plot No. 30-C, Erandawane, Pune - 411 004, Maharashtra, India

**Keywords:** Larsen Syndrome, extra carpal bones

## Abstract

Multiple carpal bones may be seen in different syndromes, especially Larsen's syndrome. A case of Larsen's syndrome with many typical features and “too many” carpal bones, is described.

An 11-year-old girl was seen by an orthopedic surgeon for treatment of knee deformities. She had kyphoscoliosis, contractures, genu valgus, hypermobility of the ankles, frontal bossing, hypertelorism, and a flat bridge of the nose. She was a dwarf. Her brother and a male cousin had similar clinical features.

Apart from other radiographs, a frontal radiograph of both hands was also obtained [Figures 1 A–D], which showed multiple (extra) carpal bones–15 on the left and 13 on the right–with abnormal lie. Additionally, negative ulnar variance was seen.

**Figure 1 (A-D):**
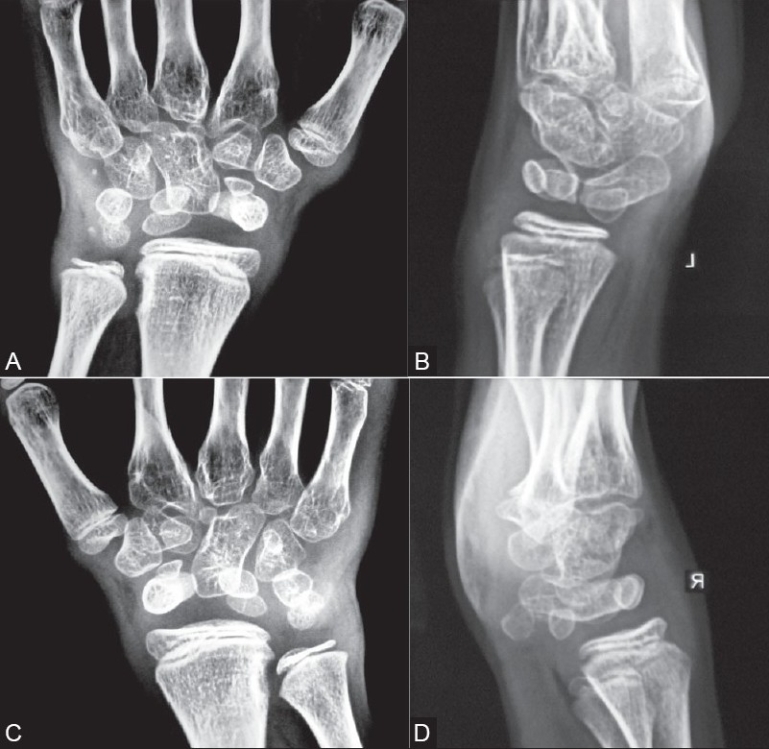
Frontal and lateral radiographs of the hands show extra carpal bones, 15 on the left (A, B) and 13 on the right (C, D), along with negative ulnar variance and impingement of the lower end of the ulna upon the medial cortex of the lower end of the radius. Note the swelling of the wrists due to the abnormal lie and position of the carpal bones

## Discussion

Extra (accessory or supernumerary ossicles) bones of the wrist have been described in syndromes such as brachydactyly A1, ulnar dimelia, hand-foot-uterus syndrome, Holt-Oram syndrome, oto-palato-digital syndrome, chondroectodermal dysplasia, diastrophic dysplasia, Gorlin's syndrome, and Larsen's syndrome.[1] As many as 20 to 25 carpal bones have been described in some cases. The other features, as described above, were typical of Larsen's syndrome.

Arthrogryposis is a descriptive term used to describe conditions with deformities due to contractures and dislocations and includes Larsen's syndrome.[2] The changes on a hand radiograph in Larsen's syndrome are typical. These include a broad or bifid thumb, polydactyly, radio-ulnar synostosis, short metacarpals, long proximal phalanges, short distal phalanges with enlarged epiphyses, and extra carpal bones.[3–5] The number of carpal bones present may vary. The patient in the present case showed 15 carpal bones on the left and 13 on the right.

In this case, the presence of extra carpal bones was a nonspecific finding, but when combined with the other characteristic features, it was suggestive of Larsen's syndrome.
